# Fabrication of one-dimensional Ag/multiwalled carbon nanotube nano-composite

**DOI:** 10.1186/1556-276X-7-195

**Published:** 2012-03-23

**Authors:** Yitian Peng, Quanfang Chen

**Affiliations:** 1 Jiangsu Key Laboratory of Design and Manufacture of Micro/Nano Biomedical Instrument, School of Mechanical Engineering, Southeast University, Nanjing, 211189, China; 2 MEMS and Nanomaterials Lab, Mechanical, Materials and Aerospace Engineering Department, University of Central Florida, Orlando, FL, 32816-2450, USA

**Keywords:** multiwalled carbon nanotube, silver composite, electroless deposition, resistance

## Abstract

Composite made of multiwalled carbon nanotubes coated with silver was fabricated by an electroless deposition process. The thickness of silver layer is about 40 to 60 nm, characterized as nano-crystalline with (111) crystal orientation along the nanotube's axial direction. The characterization of silver/carbon nanotube [Ag/CNT] nanowire has shown the large current carrying capability, and the electric conductivity is similar to the pure silver nanowires that Ag/CNT would be promising as building blocks for integrated circuits.

**PACS**: 81.05.uj, carbon nanotubes, carbon-based materials, diamond/nanocarbon composites.

## 

One-dimensional nanostructured metals play important roles as interconnects and nanoscale electronic devices. Silver nanowire is especially attractive due to its extremely high electric and thermal conductivities [1]. Template-directed synthesis and the solution-phase based approach have been widely used to produce Ag nanowires [2-4]. However, pure silver nanowires are limited for metallic interconnection applications as its resistivity increase largely with the dimension decreasing due to its increased electron scattering. On the other hand, carbon nanotubes [CNTs] are known for having long mean free path (order of several microns), extremely high current densities (> 10^9 ^A/cm^2 ^at 25°C), and high aspect ratio. The bulk composites made of metal matrix with CNTs as the reinforcement component for augmenting the metal conductivity have been extensively investigated [5-7]. However, how to fabricate and to characterize a single one-dimensional nano-composite with embedding a single CNT within the silver matrix are difficult [8,9], and electroless silver plating process has been used to deposit silver onto multiwalled carbon nanotube [MWCNT] [10]. In this paper, one-dimensional Ag/MWCNT nano-composite was prepared by coating the MWCNT with relatively uniform nano-crystalline silver layer using the electroless silver plating process. The electrical properties of one-dimensional Ag/MWCNT nano-composite were investigated using two terminal conductance measurements to demonstrate the resistivity and the current carrying capacity.

## Methods

**Figure 1:**
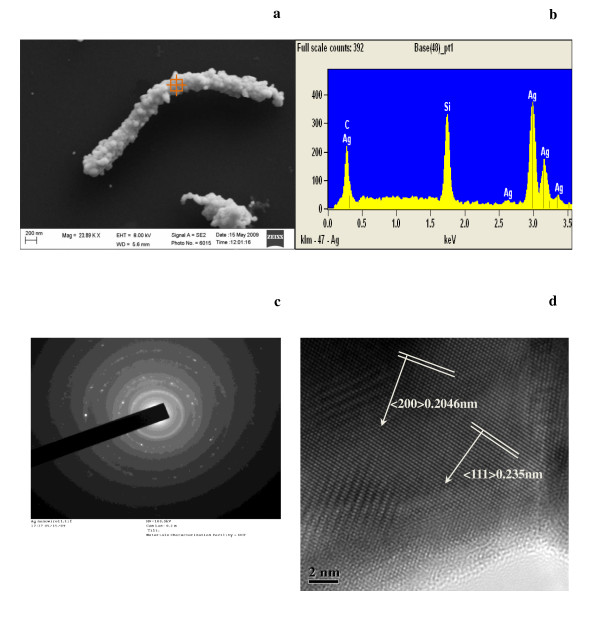
**Images of Ag/MWCNT taken by different imaging devices**. (**a**) Scanning electron microscope [SEM] image of a Ag/MWCNT that the MWCNT is completely covered by silver layer. (**b**) Energy-dispersive X-ray spectroscopy spectra of the Ag/MWCNT that no oxygen can be identified. (**c**) SAED pattern shows a multicrystal structure in Ag/MWCNT nanowire (**d**) HRTEM of the silver coated onto a MWCNT of the Ag/MWCNT nanocomposite. Arrows show the crystal orientation.

The MWCNTs used in our study were prepared by chemical vapor deposition process. The dimension of the MWCNTs is 3 to 15 μm long and the diameter ranges from 40 to 60 nm. The one-dimensional Ag/MWCNT nano-composites were fabricated using an electroless silver plating process [10-13] (see Additional file 1). Figure 1a shows that the relatively uniform and continuous silver layer was coated on MWCNT. Figure 1b reveals that the composition of the Ag/MWCNT nano-composite includes C, Si, and Ag. Silicon is from the silicon substrate. The contents of silver and carbon confirm the silver coating on the MWCNT.

## Results and discussion

Transmission electron microscopy observation shows that the one-dimensional Ag/MWCNT nano-composite has a relatively uniform silver coverage as shown in Figure 1a. The diameter is about 160 nm, with a deviation of about 20 nm, and the length is about 3.2 μm. The inset of Figure 1c is the selected area electron diffraction [SAED] pattern obtained from a massive bundle of one-dimensional Ag/silver nano-composites. Several continued rings and some diffraction spots that were observed in the SAED pattern indicate that the Ag/MWCNT nano-composites have a polycrystalline structure.

The distances of the lattice planes are measured using a high-resolution transmission electron microscopy [HRTEM] and shown in Figure 1d. Interplanar distance of the (111) planes which is 0.234 nm indicates that the nano-crystal silver coating possesses a face-centered cubic [FCC] structure, which are consistent with the standard lattice constant of the Ag crystal structure [14]. Lattice fringes with spacing of 0.2046 nm which are also observed corresponds to the (200) planes in FCC crystalline silver. It can be seen that the silver coating layer is nano-crystallized without any amorphous structure [15].

**Figure 2:**
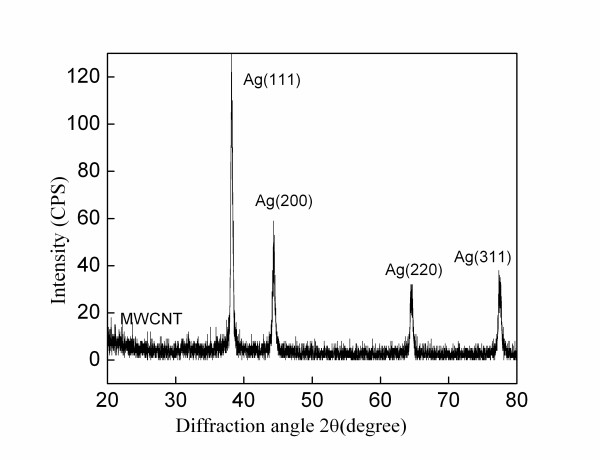
**XRD patterns of one-dimensional Ag/MWCNT nano-composite**.

Figure 2 gives the X-ray diffraction [XRD] patterns of the nano-crystalline Ag/MWCNT nano-composites. One weak peak at about 2θ = 26.00 is from MWCNTs and the peaks at 38.2°, 44.3°, 64.4° and 77.4° can be assigned, respectively, to <111>, <200>, <220>, and <311> crystalline planes of the silver coating. It is consistent with the crystalline silver measured in HRTEM. It is further observed that the nano-crystalline silver coating has a strong (111) orientation along the nanotube's axial direction because the specific free energy of silver is minimum on (111) planes of the face center cubic structure [16].

**Figure 3:**
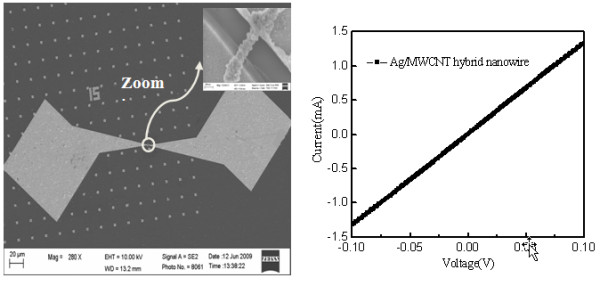
**SEM image of the setup of electrical characterization**. Inset is the magnified image of the connection (left) and the I-V curve of Ag/MWCNT nano-composite wire. The straight line indicates a resistive nature, and no semiconductive characteristics as in silver oxide can be identified.

A single, one-dimensional nano-crystalline Ag/MWCNT nano-composite wire was placed onto an electrically isolated wafer substrate, and two electrodes were made by liftoff (10 nm Cr/170 nm Au), as shown in Figure 3 (left). The gap between the two electrodes is about 500 nm and the diameter of the nano-crystalline Ag/MWCNT nano-composite is about 160 nm. Resultant electric currents were recorded under applied bias voltages, ranging from -100 to 100 mV, to the two electrodes.

Figure 3 (right) displays the typical linear I-V curves measured on the single, one-dimensional nanocrystalline Ag/MWCNT nano-composite wire at room temperature. More than five measurements were carried out on the sample. The resistance was calculated (based on the applied voltages and the resultant currents). The average resistance of the Ag/MWCNT is at about 75.7 Ω, and the variation among different measurements is less than 2%. Therefore, the resistivity of the one-dimensional Ag/MWCNT nano-composite is approximately 3.0 × 10^-6 ^Ωm. It is reasonable that the resistivity of the nanocrystalline Ag/MWCNT nano-composite is higher than the bulk silver (1.5 × 10^-8 ^Ωm) because the surface scattering of electron increases largely when the diameter is as smaller as the mean free path of the silver [16]. Since the I-V curve is a straight line, therefore, no oxidation effects can be identified. This also agrees with the composition analysis in which no oxygen was detected. It was indicated that the resistivity of a DNA-templated silver nanowire measured by SH Park et al. was about 4 × 10^-6 ^Ωm [17]. It can be deduced that the one-dimensional Ag/MWCNT nano-composites have better conductivity than the DNA-coated silver nanowire. However, the resistivity of Ag/MWCNT nanowire is greater than that of a pure single crystal silver nanowire which has the resistivity of about 1.87 × 10^-6 ^Ωm [15]. The increased resistivity of Ag/MWCNT could be due to the multigrained structure as well as the contact resistance as well. Based on the free electron theory, the electric conductivity of metallic conductors can be described as [5,18]: $\rho =\frac{ne^{2}\bar{\lambda }}{m_{\text{e}}v_{\text{F}}}$ through the free electron gas theory [5], where *n *is the electron density of state, $\bar{\lambda }$ is the mean free path, *m*_e _is the mass of electron and *V*_F _is the Fermi velocity. The electrical conductivity is proportional to the mean free path, i.e., the longer the mean free path, the higher the conductivity will be. The silver coating on the sidewalls of MWCNTs forms composite couplings to electronic states through the charge transfer interaction from the silver ions to MWCNTs [19]. The metallic MWCNTs can be ballistic conductors and have a mean free path of 0.5 μm equaling with the length of the tube [2]. The embedding MWCNT in a silver matrix facilitates electron transfer along with the ballistic transport from end to end, thereby prolonging the electron's mean free path of nano-composite ten times of the silver [20]. The low resistivity of one-dimensional Ag/MWCNT nano-composite silver nanowire can attribute to the longer mean free path of MWCNT. The current density *t *was calculated to be about 6.56 × 10^7 ^A/cm^2 ^at the voltage of 100 mV through the *t = I/S*, where *S *is the cross sectional area and *I *is the current. The current can be kept stable during the 20 min measurement under the constant voltage of 100 mV. Since electromigration occurring in Ag interconnects at the current density of 10^6 ^to 10^7^A/cm^2 ^[21], and the metallic MWCNT can stand electric current density (along axis) more than 1,000 times greater than that of silver; therefore, it is clear that integration of MWCNTs in nano-composites would be good to counter electromigration.

## Conclusions

As a summary, MWCNTs coated with about 40 to 60 nm of relatively uniform and continuous nano-crystalline silver layer were fabricated using an electroless silver plating process. The two-terminal I-V electrical measurements conducted have demonstrated that the one-dimensional nano-crystalline Ag/MWCNT nano-composite has low resistivity and high current carrying capacity at room temperature. The electrical conductivity of MWCNTs has been enhanced by depositing pure crystal silver on their surface, and the maximum current carrying capacity of silver have been enhanced by embedding with MWCNT. Therefore, the one-dimensional nano-crystalline Ag/MWCNT nano-composite wires are promising to be used as interconnects in the area of nanoelectronic device.

## Competing interests

The authors declare that they have no competing interests.

## Authors' contributions

QC has initiated the concept and awarded as PI by NSF to support this work. YP was recruited and supervised to conduct the experimental work. YP continued the work later after joining the current institution. All authors read and approved the final manuscript.

## Supplementary Material

Additional file 1 **Support 1: Fabrication of one-dimensional Ag/Multi-walled carbon nanotube nanocomposite**. Details of frbrication proicess is provided. Click here for file
