# Supplementary material for: Fabrication of one-dimensional Ag/multiwalled carbon nanotube nano-composite
Source: Nanoscale Res Lett. 2012 Mar 23;7(1):195. doi: 10.1186/1556-276X-7-195 (PMC3338368; doi:10.1186/1556-276X-7-195)
Supplement: Additional file 1 — Support 1: Fabrication of one-dimensional Ag/Multi-walled carbon nanotube nanocomposite. Details of frbrication proicess is provided. [file 1556-276X-7-195-S1.DOC]

Fabrication one-dimensional Ag/Multi-walled carbon nanotube nanocomposite

Yitian Peng, Quanfang Chen

Supporting information:

The MWCNTs were suspended in an aqueous solution of HNO_3_(70%) and maintained at 60 degrees of temperature for 2hours before doing the surface activation[11]. After this acid treatment，the MWCNTs were rinsed with de-ionized(DI) water. The sensitization was accomplished by dispersing them in a aqueous solution of 0.1M SnCl_2_/0.1M HCl for 30 min, followed by rinsing in de-ionized water. The Sn^2+^-sensitized MWCNTs were further activated in an aqueous solution of 0.014M PdCl_2_/0.25M HCl for another 30 minutes. The activated MWCNTs were washed with DI water and then introduced into electroless silver plating bath.

The electroless silver plating solution was composted of 0.005M AgNO_3_ as the ion source, 0.1M NH_3_·H_2_O as the complexant agent, and 0.1M Glyoxylic acid as the reducing agent. The PH value of the electroless silver plating solution was adjusted to 9-10 with NaOH [10]. The activated MWCNTs were introduced into the electroless silver plating cell with magnetic stirrer agitation. The plating cell was a glass beaker of 250 cm^3^ in volume and the temperature for the electroless silver deposition was kept at 60 degrees. The plating time was controlled as 10mins. The Ag/MWCNTs hybrid nanowires were then filtered and rinsed with DI water. Finally they were dried at the room temperature in a vacuum desiccator.
